# The Family Check-Up Online: A Telehealth Model for Delivery of Parenting Skills to High-Risk Families With Opioid Use Histories

**DOI:** 10.3389/fpsyg.2021.695967

**Published:** 2021-07-07

**Authors:** Elizabeth A. Stormshak, Jordan M. Matulis, Whitney Nash, Yijun Cheng

**Affiliations:** ^1^Prevention Science Institute, University of Oregon, Eugene, OR, United States; ^2^Department of Counseling Psychology and Human Services, University of Oregon, Eugene, OR, United States

**Keywords:** prevention, family, telehealth, early childhood, parenting

## Abstract

Growing opioid misuse in the United States has resulted in more children living with an adult with an opioid use history. Although an abundance of research has demonstrated a link between opioid misuse and negative parenting behaviors, few intervention efforts have been made to target this underserved population. The Family Check-Up (FCU) has been tested in more than 25 years of research, across multiple settings, and is an evidence-based program for reducing risk behavior, enhancing parenting skills, and preventing the onset of substance use. It is designed to motivate parents to engage in positive parenting practices and to change problematic parenting and has been tested across a variety of ages including early childhood and adolescence. It is highlighted in NIDA’s Principles of Substance Use Prevention for Early Childhood: A research-based guide as one of only three effective selective prevention programs for substance abuse among families with young children. Recently, we developed an online version of the FCU that has now been adapted for early childhood and families with opioid use histories. The online platform and telehealth model allow for wide-scale dissemination, ease of training with community providers, and increased public health reach for families in remote, rural areas. This is particularly important when targeting families with opioid misuse and addiction because there are high rates of addiction in remote areas, yet few services available. In this article, we describe the FCU Online and review new content in the model that targets a population of young adult parents with substance abuse histories, including opioid use. New modules include content focused on harm reduction for this high-risk population of parents, such as safety in the home, substance use while parenting, and managing conflict with partners and friends.

## Introduction

Misuse of opioids, including heroin, prescription pain relievers, and synthetic opioids, has been on the rise in the United States over the past decade, which has led to more than 400% increases in overdose death in the United States (Centers for Disease Control and Prevention, 2019). According to a 2018 national survey on drug use and health (Substance Abuse and Mental Health Services Administration, 2019), over 10 million people 12 years of age or older misused opioids, approximately 2 million people were diagnosed with opioid use disorder. Bullinger and Wing (2019) estimate about 548,000 children lived with an adult with opioid use histories in 2017. Additionally, the number of children living with an adult who uses heroin doubled from 2002 to 2017 (Bullinger and Wing, 2019). The result is that an increasing number of children are living with substance abuse in the home and more parents are taking care of their children while using substances, yet few interventions specifically target this population of parents.

Similar to patterns observed in research with parents who use a range of substances, the past research has documented the link between opioid use and negative parenting behaviors. Risk factors for negative parenting practices associated with parents with opioid use histories include psychopathology, comorbidity, socioeconomic status, parenting style or parenting knowledge, emotion regulation, level of distress, and negative care-taking behaviors (Cioffi et al., 2019). The previous studies also suggest a high level of comorbidity between opioid dependence and other mental health disorders. Furthermore, individuals with mental health disorders are more likely to be prescribed opioids, which increases the risk of opioid misuse (Goesling et al., 2015). Additionally, parents with opioid use histories are likely to receive prescription medication for mental illness and have other mental health disorder diagnoses (Novak et al., 2019), such as personality disorder (Barry et al., 2016), anxiety-related disorders (Martins et al., 2012), and depressive disorders (Sanmartin et al., 2019). Given the co-occurrence of the aforementioned mental health disorders, parents with opioid use histories often face emotion regulation challenges and high levels of distress (Neger and Prinz, 2015; Wilcox et al., 2016). For example, parents with opioid use histories often experience stages between substance craving and withdrawal, which may induce emotion regulation challenges. As both a precursor and an outcome of opioid misuse, emotion dysregulation may also impede parents’ ability to provide adequate childcare and responsive parenting (Mayes and Truman, 2002). Research also indicates that parents with opioid use histories often lack basic parenting information and utilize coercive, negative parenting strategies which exacerbate the risk of child abuse and neglect (Mayes and Truman, 2002; Cioffi et al., 2019).

Children living with an adult with opioid use histories are likely to grow up in a chaotic environment with low parental support, minimal monitoring, and high exposure to unsafe and illegal activities (Powis et al., 2000). Such settings may impede children’s ability to develop complex regulatory skills, such as emotion regulation and autonomous decision making, and increase the likelihood of engaging in risky behaviors in adolescence and young adulthood (Bridgett et al., 2015; Cioffi et al., 2019). Some research suggests a direct impact of parental substance use on child developmental outcomes while other studies argue that parental and environmental factors mediate negative long-term outcomes (Barnard and McKeganey, 2004; Pajulo et al., 2006). Key parenting skills, including maternal responsiveness and sensitivity, predict long-term positive outcomes for children even in the context of substance misuse (Lowe et al., 2017).

Parenting interventions that have been developed specifically for parents with opioid use histories focus on delivering emotional regulation skills and increasing parenting knowledge (Neger and Prinz, 2015). For example, Parents Under Pressure is a 12-session, home-based, and manualized program that aims to improve family functioning and decrease parental stress by delivering parenting skills and mindfulness techniques. The program demonstrated effectiveness in improving family functioning, reducing child problem behaviors, and preventing parental substance use relapse (Dawe et al., 2003). Niccols et al. (2012) conducted a systematic review of integrated substance abuse program that included a parenting component. While most integrated programs were associated with parenting skill improvement, reductions in maternal mental health problems, such as depression, were associated with higher levels of parenting competence.

Despite these successes, most parents with opioid use histories in the United States are not receiving any treatment, and they are rarely receiving treatment that links their substance misuse with parenting. Research shows that only 28% of adults with opioid use histories who live with a child received any type of substance use treatment within the past year (Feder et al., 2018). Additionally, parents with opioid use histories are more likely to identify barriers and stigma that prevent them from seeking proper treatment for their substance use compared to adults with opioid use histories who are not living with a child. Adults with opioid use histories who live with a child are more likely to be women than men. Historically, women are less likely to seek out substance use treatment due to unique risk profiles, such as comorbid mental health disorders and past traumatic experiences (Greenfield et al., 2007). Adults with opioid use histories who live with a child are also more likely to live in rural areas, where substance use treatment and resources focused on parenting are usually limited (Patrick and Schiff, 2017). Barriers related to accessibility, such as access to transportation and availability of childcare, are also twice as likely to be reported by parents with opioid use histories. Finally, parents with opioid use histories face a variety of stigmas that limit their ability to seek mental healthcare. Feder et al. (2018) reported that parents with opioid use histories are four times more likely to report stigma as a barrier to treatment for mental health concerns than parents without opioid use histories. Adults with opioid use histories feared being judged by neighbors or peers, removal of their children from the household, and developing a bad reputation as a parent, particularly in small rural communities.

The COVID-19 pandemic has placed additional and unprecedented barriers to treatment for parents with opioid use histories. Isolation, stress, and anxiety caused by the pandemic may increase the frequency and amount of opioid use, which can lead to exacerbation of symptoms and increased likelihood of overdose. For those who seek treatment, state and federal regulations on reducing face-to-face clinical encounters may prevent them from seeking proper care (Priest, 2020). Huskamp et al. (2020) suggest that the percentage of individuals initiating medical treatment for opioid misuse decreased during the early months of the COVID-19 pandemic. Additionally, other organizations providing services to people with opioid use histories, such as syringe service programs, have also been impacted by the pandemic. For example, Glick et al. (2020) found that 43% of 173 interviewed syringe service programs reported decreasing service provision due to the pandemic. Additionally, one-quarter of syringe service programs had one or more sites closed due to COVID-19. For people already in treatment for opioid misuse, access to any medication-assisted treatment or opioid treatment program has become one of the biggest issues. For example, patients receiving methadone were required to visit their opioid treatment program daily prior to the pandemic. While some of the requirements have been modified to allow patients to take home doses of their medication during the pandemic, this option can be challenging for some populations (Cowan et al., 2021). Some recent efforts have been made to decrease barriers for parents with opioid use histories, which will increase accessibility to treatments. For example, the (Substance Abuse and Mental Health Services Administration, 2020) recently released new guidance for patients to engage in take-home methadone maintenance programs. Agencies that serve families have increased their use of telehealth support to parents. Parenting interventions are taking important steps to decrease face-to-face contact and building programs online that reach a wide range of families.

## The Family Check-Up

The Family Check-Up (FCU) is a brief, cost-effective, and strengths-based intervention that focuses on parent management training and skill building. It relies on an ecological assessment where parents report on their current parenting strategies, child behaviors, family dynamics, and other important contextual factors including stress, social support, parenting self-efficacy, and health behaviors including substance use (Dishion and Stormshak, 2007). Following the norm-referenced assessment, parents receive a strengths-based feedback session using motivational interviewing delivered by a family consultant or coach where their assessment results are presented relative to normed data. Parents choose from a menu of options and can self-direct to additional support from their coach on topics including limit setting and monitoring, proactive parenting, positive parenting, and relationship building (Dishion and Kavanagh, 2003). The FCU is intended to be brief (three sessions) and delivered as a preventative intervention to at-risk families or it can be adapted to a tiered, targeted intervention with follow-up for high-risk families. Families engaged in the FCU experience reductions in child problem behaviors, family conflict, youth substance use, improvements in child self-regulation and academic outcomes, and increased use of parent use of positive behavior strategies. The FCU has been shown to be effective in multiple randomized control trials with a range of age groups when delivered in a variety of settings including elementary school (Stormshak et al., 2020), middle school (Stormshak et al., 2010), and community mental health settings (Smith et al., 2015). The model is effective at reducing problem behavior and supporting parenting skills with parents of young children (Dishion et al., 2008), middle school children (Fosco et al., 2013), and late adolescents (Stormshak et al., 2019a).

The FCU has a long history of efficacy trials that support this model across multiple populations; however, this research was conducted using the in-person version of the FCU, which was delivered either in the home, at a community setting, or at school. A large-scale effectiveness trial conducted in 2009 was delivered across 41 schools in Oregon, and although the results of the study were positive, there were many barriers to participation and uptake (Smolkowski et al., 2017). First, the uptake by schools was poor, with some schools unable to use the model due to staffing issues. Second, many parents faced barriers to participation, such as transportation and childcare. This led to the adaption of the FCU for online delivery (FCU Online) for parents of middle school children (Danaher et al., 2018). Parenting interventions often suffer from issues of retention and engagement, and the FCU Online removed the barrier of having to physically attend sessions and engage with the intervention at specific times. Instead, parents could access the FCU Online from their home computers and received support from a coach over the phone. Additionally, the FCU Online reduced any potential burden on school staff or community providers and can be delivered with coaching or with no support at all.

The FCU Online was developed as part of a randomized controlled trial as an approach to reduce problem behavior in middle school children (funded by the National Institute on Drug Abuse: Stormshak et al., 2019b). In the trial, the FCU Online was delivered as a stand-alone program, or with supplemental coaching in a telehealth format that included at least three phone or video conferencing sessions to support parents in making behavioral change (Danaher et al., 2018; Stormshak et al., 2019b). The FCU Online was delivered to students across eight middle schools in Oregon (both rural and urban) with a high percentage of students and families who were at risk (more than 70% economically disadvantaged and fewer than 50% passing state testing with proficiency). Results suggest that the FCU Online with coaching support improved parents’ self-efficacy (*d* = 0.25) and child emotional problems (*d* = 0.32) at 3 months post-test, with outcomes moderated by risk in the expected direction (e.g., higher risk was associated with greater improvements; Stormshak et al., 2019b). Furthermore, for children with higher levels of behavior problems, the FCU Online also showed intervention effects on effortful control and parenting confidence, key FCU mechanisms of change.

These results are promising and suggest that an online version of the FCU can be targeted at high-risk populations, such as parents who misuse opioids and other drugs. As such, we have adapted the FCU Online for parents with opioid use histories for delivery on smartphones. This will enable us to disseminate the FCU Online in rural communities—where more adults have smartphones than computers—and to have a wider reach and impact on vulnerable populations that may not have access to parenting skills interventions, or who may have stigma associated with attending these support interventions in their communities. Rural areas, particularly in Oregon, are impacted simultaneously by high rates of opioid use and lack of services for parenting support, mental health, and substance use treatment. Rural areas have been hard hit by opioid use due to easier access to prescription medications coupled with high levels of socioeconomic stress and unemployment (Keyes et al., 2014). Research clearly suggests that enhancing parenting practices when children are young can ameliorate and protect against risk factors that impact development. Ample research supports the model whereby supportive and warm parenting mediates socioeconomic status and later child problem behavior from early childhood to adolescence (Odgers et al., 2012). By adapting the FCU to an eHealth model, we address a need in the community with a cost-effective, transportable intervention focused on building parenting skills in a vulnerable population to support healthy development of children and improve overall family functioning.

## Rationale

Given the unique struggles that parents with opioid use histories face, it is important to address and support the needs of this population with evidence-based interventions. However, as outlined previously, many barriers exist in seeking treatment and parenting support. Treating substance use by integrating a family component to the intervention improves treatment engagement for substance use, as well as increases parenting skills (Sword et al., 2009; Milligan et al., 2011). Building upon our previous research and development of an eHealth version of the FCU (Danaher et al., 2018), we worked to develop an eHealth web-based mobile application of the FCU Online to allow for accessible parenting intervention within rural settings.

To ensure our intervention met the needs of our intended community, we utilized an iterative approach to intervention development, guided by family and community service focus groups, to ensure that the mobile application of the FCU Online would adequately address parenting needs. First, we conducted family focus groups of pregnant mothers with opioid use histories to identify needs of the population. Several themes emerged focused on lack of knowledge around child development, appropriate discipline practices, and behavioral routines. Additionally, difficulties in accessing services were a consistent theme throughout the focus group. Specifically, lack of knowledge navigating systems and available services, fear of judgment from providers and stigma of substance use challenges, lack of access to services, and concern about having children removed from their care. Finally, the focus group highlighted themes around flexibility in provided services, as many of the family struggled with economic instability.

Based on feedback from focus groups and community providers, we adapted the FCU model for this population of high-risk parents. First, we identified areas of content, including parent wellness and parenting in the context of substance use, that were relevant to parents with substance use histories. Once the content was identified, we used evidence-based models to develop content in each modules. Once the modules were refined, each module was then reviewed by community partners and third-party content experts to ensure their clarity and suitability to the population.

## The FCU Online for Parents with Histories of Opioid Misuse

The FCU Online is grounded in the original FCU model and includes an assessment, feedback, and curricula designed to support parents in improving their relationships with their children and building parenting skills that predict healthy long-term child adjustment. The FCU Online guides parents through an assessment, feedback, and skills training session for each module of content. At the start of each module, parents take a brief assessment where they receive feedback that identifies specific areas of strength and growth within the skill area. This assessment and feedback inform the content delivery, and the web-based or mobile application then highlights strength areas as well as areas for growth using a motivational interviewing framework.

## FCU Online Modules

The FCU Online includes five different content modules: parent wellness, substance use and parenting, positive parenting, proactive parenting, and monitoring/limit setting. The content of these modules was adapted from the Everyday Parenting curriculum (Dishion et al., 2011) and includes additional support for parent wellness and substance use prevention, which support challenges that are often associated with parents with a history of substance use. In the next section, we will briefly describe the content and how each module was specifically adapted to support parents with opioid use histories.

### Parent Wellness and Self-Care

Research suggests that individuals with opioid use histories are more likely to have higher incidences of mental health disorders, specifically anxiety and depression (Cioffi et al., 2019). Additionally, problems with self-regulation and emotional control have been shown to be both a precursor and outcome of opioid misuse, which has negative implications for parenting skills since self-regulation is needed to appropriately respond to children’s behaviors (Rutherford et al., 2015; Cioffi et al., 2019). The aim of this module is to provide psychoeducation on the importance of parent mental health and promote self-care through skills that support their child’s mental health and emotion regulation in the context of parenting.

The parent wellness and self-care module include psychoeducation and interactive activities to increase knowledge and build skills to support parent health. Specifically, the module introduces the importance of building a self-care plan to improve health and manage stress, providing suggestions and examples to choose from, if needed. The module then turns to building skills to manage depression and cope with stress, acknowledging these as common experiences for parents that have been linked with child behavior problems and are amenable to change when parents participate in parenting training and skills development (Shaw et al., 2009). The skill sessions engage parents in stress management practice through interactive activities, including behavioral activation and mindfulness techniques. The module then turns to implementing healthy routines and supporting parent wellbeing and child development. Finally, the module provides tools to improve one’s sleep routine and highlights the importance of sleep. By implementing these self-care habits and improving parent emotional control, parents can improve their ability to provide quality care for their children.

### Substance Use and Parenting

This module was designed from a harm reduction perspective since the previous research has suggested that punitive-based interventions for adults with opioid use histories are often ineffective (Taplin and Mattick, 2015). Harm reduction approaches which incorporate comprehensive treatment to address substance use within the larger context of parenting have shown success in increasing the overall health of both mothers and their children (Pinkham et al., 2012; Wright et al., 2012). Furthermore, Niccols et al. (2012) found that integrated treatment of substance use and parenting intervention for mothers was associated with improved outcomes in parenting skills compared to addiction-only treatment. The substance use module utilizes this perspective to address substance use within the context of parenting in order to support parenting development.

The module starts by engaging individuals in psychoeducation about substance use and the physiological effects on the body. Participants then practice how to manage substance cravings, by identifying triggers of use and employing strategies when experiencing cravings to build awareness. Because individuals with opioid use histories often report social isolation (Sword et al., 2009; Pinkham et al., 2012), we discuss the importance of building support through healthy relationships. We explore communication with partners and family as an essential aspect of building positive relationships (Gottman, 2008). Activities provide practice with effective communication skills, such as practicing “I” statements. Finally, the module explicitly connects the effects of substance use to parenting and emphasizes important strategies to keep children safe when substances are present in the home.

### Positive Parenting

The cornerstone of effective parenting is positive parenting, which refers to a set of skills that enable parents to guide their children using positive strategies, such as praise, incentives, and positive support. Research consistently suggests positive parenting strategies improve child behavior and mental health (Stormshak et al., 2017). Positive parenting is particularly important in early childhood, as harsh punitive parenting practices have been shown to lead to a parent-child coercion cycle, which is associated with increased child problem behavior, and more extreme problem behavior in adolescence (Smith et al., 2015). Critical tools for parents of young children involve promoting healthy development by using praise, support, and positive attention to shape behaviors; structuring activities and requests to increase the likelihood of success; and providing parent-child interaction and playtime. The FCU has been shown to improve positive parenting in high-risk families, which, in turn, disrupts the trajectory of problem behavior in young children ages 2–5 years (Dishion et al., 2008). Additionally, the previous research has shown that maternal warmth and sensitivity predict positive mother-infant dyad relationships, beyond in utero exposure to opioids (Sarfi et al., 2011). While parents with opioid use histories have fewer sensitive and warm interactions with their children compared to those without opioid use histories, interventions like the FCU that focus on relationship quality, warmth, and positive behavior have the potential to mitigate risk to parent-infant relationship quality.

The positive parenting module in the FCU Online includes interactive activities to increase awareness and attention to positive skill development. The module begins with psychoeducation around positive reinforcement (e.g., rewarding the behavior you want to see, rather than focusing on punishment). It acknowledges the challenges of raising young children and encourages parents to focus on praising good behavior rather than attending to negative behavior. In addition to encouragement and praise, the module offers opportunities for parents to practice using specific praise. Specific praise gives children explicit information about what behaviors parents want to encourage. By making praise more specific rather than global, young children can learn the behaviors that parents want to reinforce. Building high-quality relationships with children is also an important aspect of positive parenting. This module emphasizes the importance of building parent–child relationships by focusing on child strengths and engaging in child-directed play. Specific skills for playing with children are reviewed and parents are encouraged to practice these skills. Another core component of positive parenting is giving clear, age-appropriate directions to guide children’s skill development. Video examples demonstrate giving directions, praising children, and providing clear rules. Last, the module gives participants a chance to differentiate between incentives, rewards, and bribes. By identifying the differences between incentives and rewards, parents can practice implementing those skills with their children to support positive behavior at home.

### Proactive Parenting

Proactive parenting refers to a parent’s ability to plan ahead to avoid problem behaviors before they occur. Research suggests that parents’ use of proactive strategies reduces child problem behaviors and may reduce the risk of future conduct problems (Gardner et al., 2007). By utilizing proactive techniques, parents provide structure and safety for children, adding stability to facilitate a child’s success. Because parents with opioid use histories can struggle with positive or warm interactions as well as creating behavioral routines (Cioffi et al., 2019), proactive parenting also incorporates the importance of planning ahead and anticipating child behaviors.

The proactive parenting content first provides structure to help a parent identify problem events and take steps to plan any adjustments needed to avoid problems in the future. Part of proactive parenting includes setting up expectations and scaffolding learning opportunities so that children can anticipate transitions. Similar to the parent wellness module, proactive parenting emphasizes the importance of healthy routines for a child. Setting up predictable routines can help promote consistency and support overall wellbeing. Research has shown that children with consistent routines also have improved behavioral control and coping skills (Dishion et al., 2011). The module helps parents build healthy routines throughout critical points of the day, such as in the morning routine, mealtime, and at bedtime. These routines also support a healthy lifestyle through sleep, meals, and exercise. Video examples demonstrate bedtime routines and play. By engaging in the proactive parenting skills, parents can begin to incorporate small changes to provide more structure to support child success.

### Rules and Consequences

Finally, the rules and consequences module addresses how to manage behaviors by creating clear, reasonable rules with defined consequences. Adapted from the Everyday Parenting curriculum, the content focuses on limit setting and monitoring skills to keep children safe and to shape behavior. Parents who effectively implement these skills can decrease problem behaviors in children (Stormshak and Dishion, 2009). Because parents with opioid use histories may have unrealistic expectations about child behavior, incorporating skills in these areas can help parents to better understand developmentally appropriate expectations and consequences to improve parenting practices (Cioffi et al., 2019).

This module begins with clear expectations about appropriate supervision of children in early childhood. The content emphasizes the importance of consistent parenting practices, which include clear rules and directions so that children know what to expect. The module provides skills development for parents in how to create rules that are realistic and set effective limits and consequences that are developmentally appropriate for young children. These skills include content, such as ignoring and using logical consequences.

### Conclusion

Opioid use in adolescents and young adults is rising at unprecedented levels and has reached epidemic proportions in some areas of the country, particularly in rural areas. Although research on the detrimental effects of opioid use on parenting and children is relatively new, it is clear that parents with opioid use struggle with a variety of parenting skills, including positive parenting, responsivity, and consistent limit setting. Substance abuse decreases parents’ responsivity to their child, increases the chances of neglect or abuse, and prevents parents from developing the relationships with their children that are necessary for healthy child development. Parents with substance abuse histories show deficits in knowledge related to parenting and reduced pleasure in parenting their children. Parents who use opioids or have a history of use display poor relationship-building skills with their children and engage in negative parenting that leads to a range of detrimental child outcomes that begin in early childhood.

As such, to have long-term sustained effects on preventing opioid misuse in parents and to help prevent substance use and related problem behaviors in the next generation, it is critical to provide support for parenting skills to this population of parents. We have developed a version of the FCU Online for families who have a history of opioid misuse to provide parenting skills training and support to this high-risk population. Our long-term goal is to facilitate the wide-scale dissemination of the FCU Online intervention to prevent opioid misuse (i.e., prescription misuse and use of heroin and illicit synthetics) across generations by targeting parents living in rural and hard-to-reach areas who have had a history of substance misuse. Our eHealth intervention focuses on supporting parents by increasing parenting self-efficacy, stress management skills, self-regulation skills, and sleep routines, which are hypothesized to lead to the prevention of opioid misuse as well as improve mental health and increase responsive, positive parenting skills.

## Data Availability Statement

The original contributions presented in the study are included in the article/supplementary material, further inquiries can be directed to the corresponding author.

## Author Contributions

All authors listed have made a substantial, direct and intellectual contribution to the work, and approved it for publication.

### Conflict of Interest

The authors declare that the research was conducted in the absence of any commercial or financial relationships that could be construed as a potential conflict of interest.
